# Genetic Variants in the Apoptosis Gene BCL2L1 Improve Response to Interferon-Based Treatment of Hepatitis C Virus Genotype 3 Infection

**DOI:** 10.3390/ijms16023213

**Published:** 2015-02-02

**Authors:** Louise Nygaard Clausen, Nina Weis, Steen Ladelund, Lone Madsen, Suzanne Lunding, Britta Tarp, Peer Brehm Christensen, Henrik Bygum Krarup, Axel Møller, Jan Gerstoft, Mette Rye Clausen, Thomas Benfield, the DANHEP group

**Affiliations:** 1 Department of Infectious Diseases, Copenhagen University Hospital, 2650 Hvidovre, Denmark; E-Mails: ninaweis@dadlnet.dk (N.W.); tlb@dadlnet.dk (T.B.); 2 Clinical Research Centre, Copenhagen University Hospital, 2650 Hvidovre, Denmark; E-Mail: steen.ladelund.01@regionh.dk; 3 Copenhagen Hepatitis C Program (CO-HEP), Department of Infectious Diseases and Clinical Research Centre, Copenhagen University Hospital, Hvidovre and Department of International Health, Immunology and Microbiology, Faculty of Health Sciences, University of Copenhagen, 2100 Copenhagen, Denmark; 4 Department of Clinical Medicine, Faculty of Health Sciences, University of Copenhagen, 2100 Copenhagen, Denmark; 5 Department of Gastroenterology, Køge Hospital, 4600 Køge, Denmark; E-Mail: logm@regionsjaelland.dk; 6 Department of Infectious Diseases, Hillerød Hospital, 3400 Hillerød, Denmark; E-Mail: lunding@dadlnet.dk; 7 Diagnostic Center, Silkeborg Regional Hospital, 8600 Silkeborg, Denmark; E-Mail: brittarp@rm.dk; 8 Department of Infectious Diseases, Odense University Hospital, 5230 Odense, Denmark; E-Mail: peer.christensen@dadlnet.dk; 9 Department of Gastroenterology and Department of Clinical Biochemistry, Section for Molecular Diagnostics, Aalborg University Hospital, 9100 Aalborg, Denmark; E-Mail: h.krarup@rn.dk; 10 Department of Medicine, Kolding Regional Hospital, 6000 Kolding, Denmark; E-Mail: Axel.Moeller@slb.regionsyddanmark.dk; 11 Department of Infectious Diseases and Rheumatology, Copenhagen University Hospital, Rigshospitalet, 2100 Copenhagen, Denmark; E-Mail: jan.gerstoft@rh.regionh.dk; 12 Department of Hepatology, Copenhagen University Hospital, Rigshospitalet, 2100 Copenhagen, Denmark; E-Mail: metteryeclausen@hotmail.com; 13 The DANHEP group (see Appendix 1)

**Keywords:** apoptosis in HCV treatment, prediction of sustained virological response, host genetics, interferon and apoptosis interaction, spontaneous HCV resolution

## Abstract

Genetic variation upstream of the apoptosis pathway has been associated with outcome of hepatitis C virus (HCV) infection. We investigated genetic polymorphisms in the intrinsic apoptosis pathway to assess their influence on sustained virological response (SVR) to pegylated interferon-α and ribavirin (pegIFN/RBV) treatment of HCV genotypes 1 and 3 infections. We conducted a candidate gene association study in a prospective cohort of 201 chronic HCV-infected individuals undergoing treatment with pegIFN/RBV. Differences between groups were compared in logistic regression adjusted for age, HCV viral load and interleukin 28B genotypes. Four single nucleotide polymorphisms (SNPs) located in the B-cell lymphoma 2-like 1 (*BCL2L1*) gene were significantly associated with SVR. SVR rates were significantly higher for carriers of the beneficial rs1484994 CC genotypes. In multivariate logistic regression, the rs1484994 SNP combined CC + TC genotypes were associated with a 3.4 higher odds ratio (OR) in SVR for the HCV genotype 3 (*p =* 0.02). The effect estimate was similar for genotype 1, but the association did not reach statistical significance. In conclusion, anti-apoptotic SNPs in the *BCL2L1* gene were predictive of SVR to pegIFN/RBV treatment in HCV genotypes 1 and 3 infected individuals. These SNPs may be used in prediction of SVR, but further studies are needed.

## 1. Introduction

Hepatitis C virus (HCV) is a major global health issue, with 170 million individuals chronically infected and 350,000 deaths annually [1].

Apoptosis, also called programmed cell death, is one of the immune system’s responses to viral infection. Many viruses, including HCV, make use of a variety of mechanisms to neutralise interferon (IFN) secretion and block genes participating in apoptosis to escape host immune attack [2].

Apoptosis can be induced via the extrinsic or the intrinsic pathways. The intrinsic pathway, which is the aim of this study, is induced by mitochondria in response to DNA damage, oxidative stress, and viral proteins [3]. Apoptosis via the intrinsic pathway is amplified by pro-apoptotic genes (*Bax*, *Bak*, *Bad*, *Box*), whereas proteins such as B-cell lymphoma-2 (BCL2) and B-cell lymphoma 2-like 1 (BCL2L1) are anti-apoptotic. BCL2L1 and BCL2 converge at the mitochondrial permeability transition pore, which regulates the release of apoptotic regulatory proteins, such as procaspase-9 and cytochrome C [4], to inhibit apoptosis. Both the extrinsic and the intrinsic apoptosis pathways are integrated into a common final pathway resulting in caspase-dependent apoptosis [3]. Glucocorticoid-induced tumour necrosis factor (TNF) receptor-related protein ligand (GITRL) is a newly identified member of the TNF receptor superfamily [5], and its interaction with GITR enhances apoptosis via the intrinsic pathway, possibly through natural killer (NK) cell-induced inhibition of BCL2L1 [6]. We screened the pathway that initiates at the GITR ligand and via CD27-binding protein (CD27BP) terminates at the mitochondrial transmembrane proteins BCL2L1 and BCL2.

Hence, we investigated single-nucleotide polymorphisms (SNPs) in the intrinsic apoptosis pathway, hypothesising that they may influence the rates of SVR.

## 2. Results

### 2.1. Host Genotypes

We genotyped 30 SNPs in four genes (*GITRL*, *CD27BP*, *BCL2L1*, *BCL2*) (Table S1) and removed monomorphic SNPs (*n* = 10) and those in Hardy-Weinberg disequilibrium (*p*-value < 0.01 (*n* = 1, rs6121038)). Genotyping failed for 3 SNPs, leaving 16 SNPs with a call rate >95% eligible for the analysis. All SNPs showed distinct clustering. After applying an FDR threshold of <0.05, a total of 4 SNPs were found to be associated with treatment response (Table S2); all 4 were found in the *BCL2L1* gene and were in complete linkage disequilibrium (LD) with each other. Below, we report only the results of the statistically most significant SNP, rs1484994 (hereafter referred to as *BCL2L1*).

### 2.2. Treatment Responses

#### 2.2.1. Study Population and Association of *BCL2L1* with SVR and HCV Viral Load

**Figure 1:**
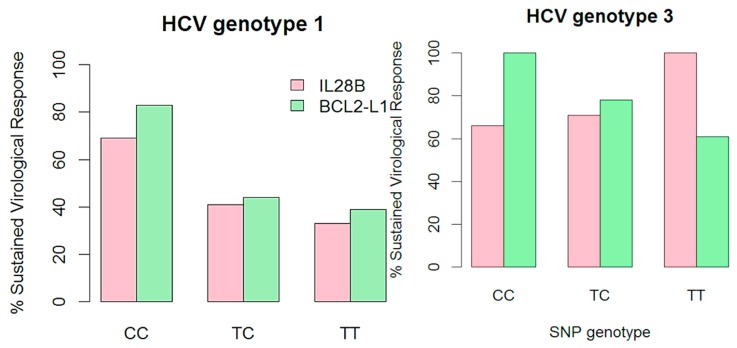
Rates of sustained virological responses according to interleukin-28B and *BCL2L1* genotypes in individuals infected with hepatitis C virus genotypes 1 or 3.

The study cohort consisted of 201 white Europeans; of these, 117 achieved SVR, and 84 failed to respond to treatment, corresponding to an overall SVR rate of 58% (95% CI; 48, 69). The main characteristics are presented in Table 1. Treatment was initiated between January 2000 and November 2009, and SVR was 46% (95% CI; 41, 51) and 70% (95% CI; 65, 75) for genotypes 1 and 3, respectively (Table 2). For HCV genotype 1, the SVR rates were significantly higher for carriers of the beneficial *BCL2L1* and *IL28B* CC genotypes compared to the non-CC genotypes (Table 2). All individuals (*n =* 5) with HCV genotype 3 that carried the *BCL2L1* CC genotype achieved SVR (Figure 1). One-hundred-and-eighty-two individuals had a measurable HCV viral load (VL) within a year of treatment initiation (20, interquartile range (IQR, 0, 64) days). For genotype 1, log_10_ HCV VL (international units (IU)/mL) was lower for carriers of the *BCL2L1* CC genotype (median, 5.9; IQR, 5.3, 6.3) compared with TC and TT carriers (median, 6.5 (IQR 5.4, 7.0); 6.5 (IQR 5.9, 6.9), *p =* 0.1), respectively. For genotype 3, HCV VL was lower for carriers of the *BCL2L1* CC genotype (median, 5.3; IQR, 5.0, 6.7) compared with TC and TT carriers (median, 6.1 (IQR 5.5, 6.4); 5.8 (IQR 5.0, 6.3), *p =* 0.7).

**Table 1:** Main characteristics of 201 individuals with chronic hepatitis C virus infection.

Characteristic	Value, *n* (%)
Male	132 (66)
HCV genotype	
1	100 (50)
3	101 (50)
Age at treatment initiation, years (median, IQR)	48 (43, 53)
HCV viral load at treatment initiation, log (IU/mL) (median, IQR)	6.1 (5.4, 6.7)
IL28B genotype, rs12979860 (*n =* 198 *)	
CC	75 (38)
CT	106 (53)
TT	17 (9)
Completion of treatment	
As scheduled	118 (59)
With dose reduction	36 (18)
Terminated before scheduled	47 (23)
Fibrosis (*n =* 102 **)	
None/light	43 (39)
Moderate/advanced	30 (32)
Cirrhosis	29 (29)
Ribavirin dose, mg/day (*n =* 199 ***)	
≤800	78 (39)
1000	57 (29)
≥1200	64 (32)
Pegylated interferon α	
2a	133 (66)
2b	68 (34)

HCV, hepatitis C virus; IQR, interquartile range; IL28B, interleukin 28B. * Three individuals could not be genotyped for IL28B; ** 99 individuals had missing information regarding fibrosis; *** one individual did not receive ribavirin.

**Table 2:** Comparison of sustained virological treatment response rates in 201 individuals with chronic hepatitis C virus infection.

Characteristic	HCV Genotype 1, *n* = 100			HCV Genotype 3, *n* = 101		
	SVR *n =* 46 (46%)	Non-Response, *n =* 54 (54%)	*p*-Value	SVR, *n =* 71 (70%)	Non-Response, *n =* 30 (30%)	*p*-Value
Sex			0.7			1
Female	16 (50)	16 (50)		26 (70)	11 (30)	
Male	30 (44)	38 (56)		45 (70)	19 (30)	
Age at treatment initiation			0.2			0.03
<40 years	14 (58)	10 (42)		36 (82)	8 (18)	
≥40 years	32 (42)	44 (58)		35 (61)	22 (39)	
HCV viral load at treatment initiation *			0.3			0.06
<5.8 log_10_ IU/mL	16 (53)	14 (47)		38 (79)	10 (21)	
≥5.8 log_10_ IU/mL	30 (43)	40 (57)		33 (62)	20 (38)	
*IL28B* genotype			0.01			0.2
CC	19 (70)	8 (30)		32 (67)	16 (33)	
TC	22 (37)	37 (63)		33 (70)	14 (30)	
TT	3 (27)	8 (73)		6 (100)	0	
*BCL2L1* genotype			0.01			0.2
TT	17 (36)	30 (64)		35 (65)	19 (35)	
TC	15 (41)	22 (59)		31 (74)	11 (26)	
CC	10 (83)	2 (17)		5 (100)	0	

Non-response comprises non-responders and relapsers. HCV, hepatitis C virus; SVR, sustained virological response; *IL28B*, interleukin 28B rs12979860 single-nucleotide polymorphism (SNP); *BCL2L1*, B-cell lymphoma 2-like 1, rs1484994 SNP. * HCV RNA viral load was not measured within 365 days of treatment initiation for 10 individuals.

#### 2.2.2. Logistic Regression Analysis of *BCL2L1* Association to SVR

The distribution of the *BCL2L1* C alleles indicated a dominant effect of the C allele on SVR for both HCV genotypes 1 and 3 (Table 2 and Table S3); therefore, the CC and TC genotypes were combined. In a multivariate logistic regression stratified by HCV genotype, the *BCL2L1* CC + TC genotype was associated with a 3.4 higher OR in SVR for HCV genotype 3 (*p =* 0.02) (Table 3). The effect estimate was similar for genotype 1, but the association did not reach statistical significance. For genotype 1, sex, age, and HCV VL at treatment initiation were not significantly associated with SVR. For genotype 3-infected individuals, age < 40 years at treatment initiation (adjusted odds ratio (aOR), 3.1 (1.1, 9.2)) and HCV VL < 5.8 log_10_ IU/mL (aOR, 3.4 (1.1, 10.7)) were associated with SVR. All four *BCL2L1* SNPs associated with SVR in the initial analyses, remained significantly associated with SVR in the multivariate logistic regression with effect estimates similar to rs1484994. Of note, this is not surprising as the four SNPs are in complete linkage with each other.

**Table 3:** Multivariate logistic regression of sustained virological response in 182 individuals with chronic hepatitis C virus infection.

Characteristic	HCV Genotype 1 (*n =* 91)		HCV Genotype 3 (*n =* 91)	
	Adjusted Odds Ratio (95% CI)	*p*-Value	Adjusted Odds Ratio (95% CI)	*p*-Value
*IL28B,* rs12979860		0.01		0.6
TT	1		1	
TC	0.97 (0.2, 4.9)		0.4 (0, 3.1)	
CC	5.7 (1.0, 32.5)		0.4 (0, 3.4)	
*IL28B*, rs12979860		0.002		0.8
TC + TT	1		1	
CC	5.9 (1.9, 17.3)		1.1 (0.4, 3.4)	
*BCL2L1*, rs1484994		0.049		0.06
TT	1		1	
TC	1.5 (0.5, 4.3)	0.4	2.8 (0.9, 9.7)	0.08
CC	9.0 (1.6, 52.3)	0.01	2.9 (0.5, infinity)	0.4
*BCL2L1*, rs1484994		0.1		0.02
TT	1		1	
TC + CC	2.2 (0.8, 5.9)		3.4 (1.2, 9.8)	

The model was adjusted for age and HCV viral load at treatment initiation and *IL28B* genotypes. Abbreviations: HCV, hepatitis C virus; CI, confidence interval; *IL28B*, interleukin 28B; *BCL2L1*, B-cell lymphoma 2-like 1.

### 2.3. Ability of Gene- and Non-Gene Classifiers to Predict Treatment Response

We constructed receiver operating characteristic (ROC) curves for factors associated with SVR (Figure S1). We defined four curves consisting of factors known to predict SVR to evaluate the non-genetic *vs.* genetic contribution in SVR prediction: the non-gene classifier consisted of the viral and demographic variables HCV VL and age, the two-gene classifier consisted of *IL28B* and *BCL2L1* SNPs, the combined IL28B*non-gene classifier consisted of the *IL28B* SNP, HCV VL and age variables and the two gene*non gene classifier consisted of *IL28B*, *BCL2L1*, HCV VL and age.

In individuals with HCV genotype 1, the curves of the non-gene classifier had an area under the curve (AUC) of 0.60, and the curves of the two-gene classifier had an AUC of 0.74. When combining the non-gene classifier with the two-gene classifier, the AUC increased to 0.79 (*p =* 0.003). In individuals with HCV genotype 3, the genetic effect appeared to be attenuated. The AUC increased from 0.71 for the non-gene classifier to 0.79 for the two-gene classifier *none-gene classifier (*p =* 0.3). The *p*-values refer to comparison with the non-gene classifier.

## 3. Discussion

### Identification of SNPs in the BCL2L1 Gene that Are Predictive of SVR to pegIFN/RBV Treatment of Genotype 3 Chronic Hepatitis C

Our findings, although preliminary, suggest a role for apoptosis in the pegIFN/RBV-mediated elimination of HCV. Interestingly, individuals achieving SVR have been shown to have more apoptotic activity prior to and during pegIFN/RBV treatment compared to individuals with non-response or relapse [7,8]. In line with this, Anatol *et al.* [9] reported that individuals with non-response to HCV treatment might have higher levels of expression of the anti-apoptotic gene *BCL2*. Shaker *et al.* [10] showed an association between an SNP (rs1800477) in *BCL2* and SVR in individuals infected with HCV genotype 4. The variant genotype was found to double SVR compared to individuals with the common genotype. Unfortunately, we have repeatedly been unable to genotype rs1800477. Given that IFN stimulates apoptosis and that we observed an effect on SVR for the variant allele of polymorphisms in *BCL2L1*, we suggest that the degree of IFN-induced apoptosis may be influenced by polymorphisms in anti-apoptotic genes, as shown by us and others [10]. Functional studies will be required to clarify how such polymorphisms may affect the response to treatment.

The treatment paradigm of chronic hepatitis C (CHC) has changed dramatically with the development of direct-acting antivirals (DAAs) targeting different parts of the HCV lifecycle. Interestingly, the NS5B polymerase inhibitor sofosbuvir, combined with RBV and pegIFN, appears to have less activity towards genotype 3 than genotypes 1 and 2 [11,12,13]. The clinical implication of our findings may be the ability to predict SVR in individuals infected with HCV genotype 3, for whom *IL28B* has no predictive value. This would allow the better targeting of PEG-IFN/RBV treatment, thus avoiding its unpleasant side effects, and reducing the overall treatment costs. Indeed, a genetic predictor of SVR in this genotype may be helpful when selecting the best treatment strategy. Further, given the high cost of DAAs, pegIFN and ribavirin are expected to remain the standard of care throughout most of the world for many years.

Our study was not designed to clarify whether the identified SNPs upregulate or downregulate apoptotic activity during HCV treatment; nonetheless, we speculate that the polymorphisms in the *BCL2L1* gene alter the expression of the anti-apoptotic protein in favour of SVR. Pathogenic changes in genes are often caused by small-scale sequence changes in either the coding sequence or regulatory regions of a gene. All SNPs identified here are synonymous non-coding variants located within a region of 51 kb in intron 1. Additionally, we genotyped all known non-synonymous SNPs in the gene, but all were monomorphic. Thus, non-synonymous genetic variation could not explain the differences in outcome. It is unlikely that a single base change in an intronic region may alter the configuration of the gene product and the function of the protein. Therefore, we speculate that the SNPs identified may be in linkage with genetic variation at restriction sites, transcriptions sites in the promoter region, or in splicing sites. This was recently shown for the *IL28B* SNP rs12979860, which was found to be strongly linked to a genetic variant encoding a new interferon protein (interferon lambda 4). It was suggested that IFN-lambda 4 is a less potent IFN that lowers responsiveness to treatment with interferon-α [14]. Furthermore, non-coding SNPs may affect the expression of *BCL2L1* by interfering with microRNA (miR). Guo *et al.* [15] showed that miR-16 is involved in the regulation of apoptosis in activated hepatostellate cells by interfering with the expression of *BCL2* and thereby mediating resistance to apoptosis.

Interestingly, the predictive value of *BCL2L1* or *IL28B* exceeded the predictive value of non-genetic factors. Furthermore, the addition of the information conferred by the *BCL2L1* polymorphism to the *IL28B* polymorphism resulted in a significant increase in the AUC for HCV genotype 1. As the introduction of DAAs in HCV treatment attenuated the impact of *IL28B* on SVR prediction [16,17,18,19], it is of interest to assess the impact of other host genetic predictors, such as *BCL2L1*, on SVR in triple therapy. Of note, the four pivotal GWASs on treatment-induced HCV clearance used seven different SNP genotyping arrays [20,21,22,23]. The *BCL2L1* SNP rs1484994 is present on the Illumina Human1M-duo array used by Rauch *et al.* [21]. However, neither the rs1484994 SNP nor other SNPs in the *BCL2L1* gene are among the top-20 SNPs reported to have significant association with SVR in HCV treatment.

The strengths of our study include the nationwide multi-centre design and population-based inclusion of study subjects. Moreover, our results were robust when corrected for multiple testing; however our study also has limitations. Despite the importance of fibrosis in predicting SVR, we were unable to adjust our analyses for fibrosis due to inconsistent reporting. Sensitivity analyses restricted to individuals with available information on fibrosis (*n =* 102) showed similar estimates of the effect of *BCL2L1*. The logistic regression was limited to the 182 individuals with available HCV VL within one year of treatment initiation. Our sensitivity analyses revealed a similar effect of *BCL2L1*, regardless of the availability of HCV VL measurements. We included patients with a minimum of 12 weeks of treatment to ensure that only non-responders with adequate drug exposure were evaluated. In the sensitivity analyses, the SVR estimates were comparable when we increased the minimum duration of treatment to 24 weeks.

In conclusion, our study suggests a role for polymorphisms in an apoptosis gene in the pegINF/RBV-mediated elimination of HCV. Our findings are preliminary and require replication in other cohorts and with treatment regimens including DAAs to further investigate the potential of this apoptosis gene in treatment response prediction.

## 4. Experimental Section

### 4.1. Study Subjects

Individuals were included from The Danish Database for Hepatitis B and C (DANHEP). DANHEP is a nationwide cohort study with ongoing enrollment and has previously been described [24]. Briefly, it contains demographic, clinical, and laboratory information on persons older than 16 years admitted to hospital with chronic hepatitis B virus (HBV) or HCV infection and who have been seen at least once after 1 January 2002, in one of the 16 medical departments that monitors and treats individuals with chronic hepatitis in Denmark. Inclusion criteria were (a) CHC defined as remaining HCV RNA positive for >6 months and (b) treatment naive individuals who had initiated treatment with pegylated interferon-α (PegIFN)/ribavirin (RBV) and with available blood samples for DNA extraction. Exclusion criteria were a positive HBV or HIV test, non-white European origin [25], participation in clinical trials, or inability to complete the first 12 weeks of treatment. Individuals were treated with pegIFN/RBV according to national guidelines [26,27,28]. Briefly, pegylated interferon α (2a/2b) was administered weekly according to the manufacturer’s instructions; RBV was given daily, adjusted for body weight for HCV genotype 1, or as a “flat dosage” for genotype 3, according to the manufacturer’s instructions. The treatment duration was planned for 48 or 24 weeks for genotypes 1 or 3 respectively. In general, if HCV-RNA titres had not decreased by a minimum of 2 log values after 12 weeks, treatment was stopped according to international guidelines. SVR was defined as undetectable plasma HCV RNA at 24 weeks after treatment cessation. Relapse response (RR) was defined as undetectable HCV RNA during treatment but detectable in the follow-up period. Non-response (NR) was defined as detectable HCV RNA throughout treatment. The study was approved by the Danish Data Protection Agency (J.No. 2011-331-0514).

### 4.2. SNP Selection and Genotyping

SNPs were selected based on the following criteria: (1) all non-synonymous SNPs with known frequencies in a white European population; (2) synonymous SNPs located in the coding region at a minor allele frequency (MAF) >5% in a white Europeanpopulation; (3) synonymous SNPs located outside the coding region at an MAF >25% in a white European population from dbSNP [4]. SNP genotyping was performed in two rounds; after the initial genotyping according to the above-mentioned criteria, we performed another search for non-synonymous SNPs and included an additional 12 non-synonymous SNPs that were genotyped in 187 individuals for whom DNA was still available. The SNP genotyping was performed by KBioscience (KBioscience, Hertfordshire, UK) using a competitive allele-specific PCR [29].

### 4.3. Statistical Analyses

SNP frequencies were compared using the Chi^2^ test or Fisher’s exact test, as appropriate. A false discovery rate (FDR) threshold of <0.05 was used to correct for multiple testing to minimise false-positive associations, corresponding to a significance level <0.003 for the highest ranking *p*-value (Table S1). SNPs fulfilling the criteria of an FDR < 0.05 were further considered in logistic regression analyses, which were adjusted for factors known to affect the rate of SVR (age and HCV viral load (VL) at treatment initiation and *IL28B*). For determination of the ability of new SNPs to discriminate individuals with SVR from those without, we constructed receiver operating characteristic (ROC) curves and presented the areas under the curves (AUC) with 95% CI. We stratified all analyses according to HCV genotype. Relapsers and non-responders were merged into one non-response group for the analysis. HCV VL was log_10_ transformed for the analysis. The results are presented using odds ratios (ORs) and adjusted ORs (aORs) with 95% confidence intervals (CIs). LD analysis was performed using Haploview 4.2 (Broad Institute of Harvard and MIT, Cambridge, MA, USA). All data were analysed using R (v.2.15.1, R: A language and environment for statistical computing. R Foundation for Statistical Computing, Vienna, Austria) or Statistical Analysis Systems (SAS v. 9.3; SAS Institute, Cary, NC, USA).

## 5. Conclusions

In conclusion, our study suggests a role for polymorphisms in an apoptosis gene in the pegINF/RBV-mediated elimination of HCV. Our findings are preliminary and require replication in other cohorts and with treatment regimens including DAAs to further investigate the potential of this apoptosis gene in treatment response prediction.

## Supplementary Materials

Supplementary materials can be found at http://www.mdpi.com/1422-0067/16/02/3213/s1.

## Appendix

**Appendix 1:** Members of the DANHEP group. Nina Weis, Department of Infectious Diseases, Copenhagen University Hospital, Hvidovre; Toke Barfod, Department of Infectious Diseases, Roskilde Hospital; Jens Bukh, Copenhagen Hepatitis C Program (CO-HEP), Department of Infectious Diseases and Clinical Research Centre, Copenhagen University Hospital, Hvidovre and Department of International Health, Immunology, and Microbiology, Faculty of Health Sciences, University of Copenhagen; Peer Brehm Christensen, Department of Infectious Diseases, Odense University Hospital; Mette Rye Clausen, Department of Hepatology, Copenhagen University Hospital, Rigshospitalet; Jan Gerstoft, Department of Infectious Diseases and Rheumatology, Copenhagen University Hospital, Rigshospitalet; Karin Groenbæk, Department of Gastroenterology, Copenhagen University Hospital, Hvidovre; Jesper Bach Hansen, Department of Gastroenterology, Aalborg University Hospital; Henrik Bygum Krarup, Department of Gastroenterology and Department of Clinical Biochemistry, Section for Molecular Diagnostics, Aalborg University Hospital; Kim Krogsgaard, The Brain Prize, COBIS; Alex Lund Laursen, Department of Infectious Diseases, Aarhus University Hospital, Skejby; Jens Lindberg, Department of Internal Medicine, Herning Regional Hospital; Suzanne Lunding, Department of Infectious Diseases, Hilleroed Hospital; Lone Madsen, Department of Internal Medicine, Koege Hospital; Axel Moeller, Department of Internal Medicine, Kolding Regional Hospital; Henrik Nielsen, Department of Infectious Diseases, Aalborg University Hospital; Jens Ole Nielsen, Department of Infectious Diseases, Copenhagen University Hospital, Hvidovre; Peter Noerregaard, Department of Internal Medicine, Frederiksberg Hospital; Ove B. Schaffalitzky de Muckadell, Department of Gastroenterology, Odense University Hospital; Poul Schlichting, Department of Gastroenterology, Herlev University Hospital; Britta Tarp, Diagnostic Center, Silkeborg Regional Hospital; Reimar W. Thomsen, Department of Clinical Epidemiology, Aarhus University Hospital.
